# Developments and Future Directions in Stretchable Display Technology: Materials, Architectures, and Applications

**DOI:** 10.3390/mi16070772

**Published:** 2025-06-30

**Authors:** Myung Sub Lim, Eun Gyo Jeong

**Affiliations:** 1 School of Electrical Engineering, Korea Advanced Institute of Science and Technology (KAIST), Daejeon 34141, Republic of Korea; 2 Department of Electronics Engineering, Incheon National University (INU), Incheon 22012, Republic of Korea

**Keywords:** stretchable displays, flexible electronics, wearable healthcare, energy-autonomous systems, durable encapsulation, artificial intelligence integration, high-resolution displays, soft robotics

## Abstract

Stretchable display technology has rapidly evolved, enabling a new generation of flexible electronics with applications ranging from wearable healthcare and smart textiles to implantable biomedical devices and soft robotics. This review systematically presents recent advances in stretchable displays, focusing on intrinsic stretchable materials, wavy surface engineering, and hybrid integration strategies. The paper highlights critical breakthroughs in device architectures, energy-autonomous systems, durable encapsulation techniques, and the integration of artificial intelligence, which collectively address challenges in mechanical reliability, optical performance, and operational sustainability. Particular emphasis is placed on the development of high-resolution displays that maintain brightness and color fidelity under mechanical strain, and energy harvesting systems that facilitate self-powered operation. Durable encapsulation methods ensuring long-term stability against environmental factors such as moisture and oxygen are also examined. The fusion of stretchable electronics with AI offers transformative opportunities for intelligent sensing and adaptive human–machine interfaces. Despite significant progress, issues related to large-scale manufacturing, device miniaturization, and the trade-offs between stretchability and device performance remain. This review concludes by discussing future research directions aimed at overcoming these challenges and advancing multifunctional, robust, and scalable stretchable display systems poised to revolutionize flexible electronics applications.

## 1. Introduction

The advancement of display technology has continuously progressed toward achieving greater mechanical flexibility to meet the evolving demands of wearable electronics, biomedical devices, and next-generation human–machine interfaces. While rigid and later flexible displays have enabled new form factors through bending and folding capabilities, they fall short in applications that require mechanical stretchability—a key parameter for conformability to nonplanar, dynamic, and deformable surfaces.

In particular, applications such as skin-mounted electronics, clothing-integrated systems, and implantable devices demand displays that can stretch, twist, and compress while maintaining optical and electrical performance. In these scenarios, conventional flexible displays are inherently limited due to their inability to accommodate strain beyond simple curvature. Consequently, the emergence of stretchable displays represents a necessary evolution rather than an optional enhancement in the display paradigm.

Notable progress has been made in this area through pioneering studies across multiple application domains. For instance, skin-attachable stretchable displays, as demonstrated by the John A. Rogers group, have enabled conformal electronic skin (e-skin) systems that mimic the mechanical properties of human tissue [1]. In parallel, textile-integrated displays, developed by Takao Someya and Tae-il Kim, illustrate the seamless incorporation of displays into fabrics, offering promising routes toward next-generation wearable platforms [2,3]. Furthermore, implantable stretchable electronics, including displays and substrates engineered for reliable operation on dynamic biological surfaces, have shown promising functionality in systems that mimic skin or attach to organs like the heart [4]. In addition to these biomedical applications, stretchable devices have also been implemented in soft robotics and medical rehabilitation tools, where their ability to conform to nonplanar, deformable surfaces and maintain electrical performance plays a critical role [5,6].

These developments highlight the transformative potential of stretchable displays in enabling intimate integration with the human body and responsive environments. However, achieving reliable stretchability in displays requires not only innovation in materials but also strategic approaches in mechanical design, system integration, and fabrication techniques. Unlike traditional display technologies that rely on mechanical rigidity to maintain structural stability and performance, stretchable displays must endure frequent and multidirectional deformation while preserving uniform luminance, resolution, and durability. This necessitates a holistic approach that simultaneously addresses material properties, mechanical architecture, and interface engineering.

To address these multifaceted challenges, stretchable display technologies are generally categorized into three main types based on their underlying strategies. The first category is intrinsic stretchable displays, which utilize inherently deformable materials such as elastomeric substrates, stretchable electrodes (e.g., silver nanowires, carbon nanotubes), and compliant emissive layers including polymer-based light-emitting materials. These displays achieve uniform stretchability by ensuring that all functional components can undergo mechanical deformation without fracture or performance degradation. The second category, wavy surface displays, employs geometrically engineered structures such as buckled films or wrinkled surfaces to accommodate strain through out-of-plane deformation. This approach allows mechanical stress to be distributed away from active layers, enabling stretchability without the need for intrinsically deformable materials. Finally, hybrid stretchable displays integrate both material innovations and structural design techniques to optimize mechanical compliance and device robustness. In particular, serpentine architectures—which incorporate sinusoidal or wavy interconnects into stretchable materials—represent a prominent hybrid design, offering high stretchability while maintaining electrical continuity. Other hybrid strategies include pre-strained configurations, multilayer stacking, and patterned composite layouts, all of which are increasingly being employed in high-performance prototypes.

This review provides a comprehensive survey of the most significant research works from the past two decades across these three categories. We highlight the key design strategies, material systems, and device-level demonstrations, while also identifying current challenges and future opportunities in the field of stretchable display technology. By synthesizing insights from material science, mechanical engineering, and electronics integration, we aim to provide a foundational understanding and a forward-looking perspective on the future of stretchable display systems.

To complement this structural categorization, we also consider key performance metrics that are commonly used to evaluate stretchable displays. To enable meaningful comparison and analysis across diverse stretchable display technologies, it is essential to establish a consistent framework for performance evaluation. Among the most commonly employed metrics is maximum stretchability, which refers to the highest strain a device can withstand without functional degradation. Mechanical durability is typically assessed through cyclic stretching tests, evaluating whether the optical and electrical properties remain stable under repeated deformation. Additional key parameters include optical stability (e.g., luminance retention), electrical reliability (e.g., resistance variation), resolution preservation, and luminance uniformity under strain.

For practical applications, environmental stability—such as resistance to humidity, temperature fluctuations, and bio-related factors like sweat—also plays a crucial role. Furthermore, interfacial robustness between heterogeneous components, particularly in multilayered or hybrid systems, is critical to ensure long-term operational integrity by preventing delamination or microcracking. In more advanced systems, secondary performance indicators such as response speed, power consumption, spatial resolution, and even user-perceived comfort (e.g., skin-conformability) may also be relevant, depending on the intended use case.

While applying a uniform set of evaluation criteria to all display technologies is often impractical, this review highlights the most representative performance aspects of each approach when particularly relevant. By focusing on key functional metrics that best characterize each strategy, we aim to provide insights that go beyond a descriptive summary, clarifying not only the current technological landscape but also the trade-offs and future directions within the field.

## 2. Categories of Stretchable Display Technologies

### 2.1. Intrinsic Stretchable Displays

Intrinsic stretchable displays rely on the use of materials that are inherently capable of withstanding mechanical deformation, such as stretching, bending, or twisting. Unlike structural strategies that depend on geometry to absorb strain, intrinsic approaches focus on engineering stretchable conductors, light-emitting layers, and substrates at the material level. This allows for compact, fully deformable displays without introducing significant topographical complexity. However, material synthesis, device integration, and trade-offs in electrical and optical performance remain central challenges. The following subsections explore stretchable electrodes, emissive layers, and fully integrated systems based on intrinsically deformable materials.

#### 2.1.1. Stretchable Electrodes

The development of intrinsically stretchable electrodes is a foundational requirement for the realization of truly deformable optoelectronic devices, including stretchable displays. Unlike conventional electrodes—such as indium tin oxide (ITO), thin metal films, or rigid conductive oxides—that readily fracture under tensile deformation, stretchable electrodes must exhibit high conductivity under significant strain, often exceeding 50%, while remaining compatible with active display components. These demands have led to the exploration of diverse classes of materials, including one-dimensional nanostructures, two-dimensional materials, liquid metals, and composite-based hybrids.

**Figure 1:**
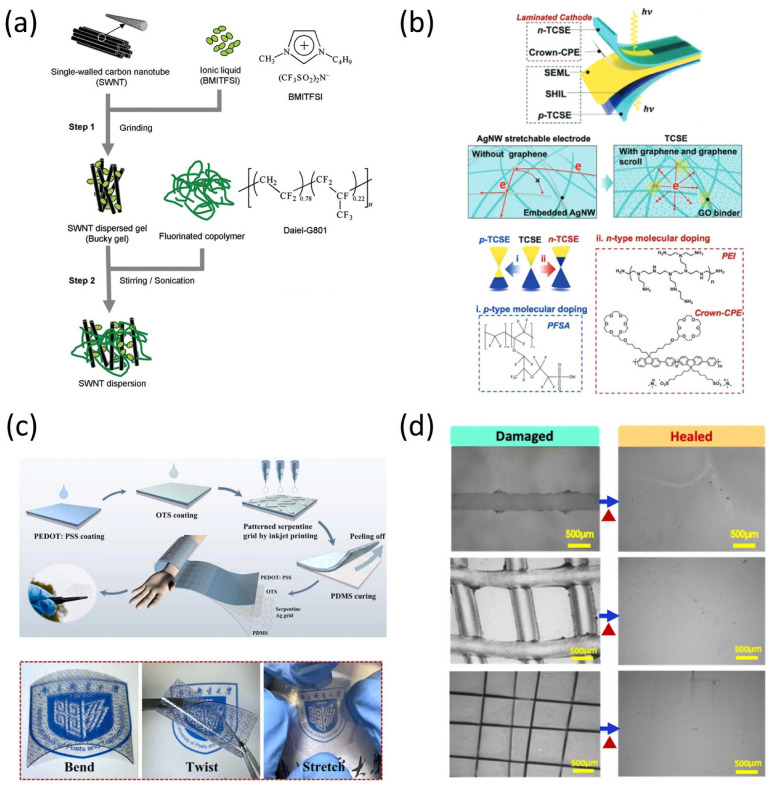
Representative strategies for intrinsic stretchable electrodes. (**a**) Manufacturing process of SWNT film, SWNT elastic conductor, and SWNT paste. Reprinted with permission from Ref. [7]. © 2008, AAAS. (**b**) Conceptual illustration of the ISOLED based on p- and n-doped 2D-contact stretchable electrodes (TCSEs), and schematic of pristine silver nanowire (AgNW) and TCSE structure. Reprinted with permission from Ref. [8]. © 2022, Wiley-VCH GmbH. (**c**) Schematic illustration of the fabrication procedures of PEDOT:PSS/S-Ag/PDMS hybrid electrodes. Reprinted with permission from Ref. [9]. © 2025, Elsevier. (**d**) Optical microscope images of PEDOT:PSS/PU/PEG film after scratching with a pencil and healing: single scratching and grid scratching. Reproduced from Ref. [10]. © 2024 The Royal Society of Chemistry. Licensed under CC BY 3.0.

A pioneering contribution to this field was made by Sekitani et al., who demonstrated a rubberlike stretchable active matrix constructed from organic transistors and elastic conductors capable of sustaining up to 70% biaxial strain [7]. This study not only validated the concept of mechanically compliant interconnects but also introduced structural design principles that decouple mechanical strain from the electrical pathway. Building on this foundation, subsequent studies focused on tuning the microstructure of electrode networks to ensure conductivity retention during deformation. One example involves the use of single-walled carbon nanotube (SWNT)-based conductors fabricated through scalable processes (Figure 1a).

Among material classes, carbon-based nanomaterials—such as carbon nanotubes (CNTs) and graphene—have gained significant traction due to their inherent flexibility and high aspect ratios. In 2022, a study introduced graphene-based intrinsically stretchable 2D-contact electrodes, enabling charge injection under strain without compromising optoelectronic device efficiency [8]. These electrodes, based on both p- and n-type doping, achieve excellent mechanical compliance and electrical performance (Figure 1b). These materials can be dispersed within elastomeric matrices or structured into serpentine mesh networks to facilitate multi-axial stretchability, though issues such as junction resistance and mechanical fatigue under cyclic strain remain challenging.

Metal nanowire networks, particularly those composed of silver nanowires (AgNWs), represent another viable approach. While AgNWs offer high conductivity and transparency, they are prone to crack propagation at high strain levels. To address this, hybrid systems—such as metallic grids embedded in stretchable substrates—have been proposed. Liu et al. demonstrated that patterned metal grids embedded in polydimethylsiloxane (PDMS) maintain conductivity under mechanical deformation and are suitable for display applications requiring optical clarity and stretchability [9] (Figure 1c).

In addition to traditional stretchable conductors, recent developments in self-healing and recyclable electrode materials offer promising solutions for long-term reliability and environmental sustainability. Kim et al. introduced a conductive blend combining PEDOT:PSS with custom-designed polyurethane and polyethylene glycol, achieving exceptional stretchability (~350% strain), mechanical toughness (~24.6 MJ/m^3^), and moderate conductivity (~10 S/cm), while maintaining electrical performance even after 20 recycling cycles and repeated self-healing [10]. This multifunctional electrode material exemplifies how future stretchable electronics can simultaneously meet performance and sustainability requirements. A representative schematic of this self-healing electrode material is illustrated in Figure 1d, which highlights the hydrogen-bonding interactions among PEDOT:PSS, polyurethane, and PEG that enable autonomous recovery of both mechanical and electrical properties.

While intrinsic stretchable electrodes can inherently accommodate mechanical deformation, in cases where such materials are not employed, mechanical design becomes critically important. To effectively distribute strain and minimize localized stress concentrations, serpentine, horseshoe, and fractal geometries have been widely adopted. For example, Kim et al. developed stretchable microelectrode arrays incorporating mechanical decoupling layers and ultra-soft elastomers, enabling stable signal transduction under repeated strain conditions [11]. Such strategies are particularly advantageous in epidermal display applications, where frequent bending, compression, and torsion are inevitable. This section serves as a brief overview of mechanical approaches in the absence of intrinsically stretchable materials; more detailed discussions and representative studies will be introduced in the following sections.

Despite these advances in the intrinsic electrode, several technical barriers remain. One of the primary concerns is the stability of the electrode under cyclic strain over extended periods. Electromechanical fatigue, interfacial delamination, and time-dependent creep can degrade both performance and lifetime. Additionally, trade-offs between conductivity, transparency, and stretchability often force compromises that limit the scalability of proposed systems. Future research will likely focus on multi-material hybrid systems that synergistically combine different mechanical and electrical properties, along with novel fabrication techniques such as inkjet printing, spray coating, or roll-to-roll processing tailored for stretchable substrates.

In summary, the field of stretchable electrodes has evolved from proof-of-concept prototypes to advanced material systems with strong potential for integration into commercial-grade stretchable displays. Continued innovation in materials, structural design, and processing methods will be critical to overcoming the remaining challenges and unlocking widespread application across wearable electronics, biomedical diagnostics, and next-generation user interfaces.

#### 2.1.2. Stretchable Emissive Layers

A key technical challenge in stretchable display research is the development of intrinsically deformable emissive layers that maintain high optical efficiency under mechanical strain. In conventional display architectures, emissive components—such as small molecule or polymer-based organic light-emitting diodes (OLEDs), quantum dots, and perovskites—are typically deposited on rigid or slightly flexible substrates and encapsulated within multilayer stacks. When applied to stretchable platforms, however, these brittle or delamination-prone layers suffer significant performance degradation under tension or compression. Therefore, enabling stretchability at the level of the light-emitting layer itself is essential for achieving full mechanical compliance in display devices.

**Figure 2:**
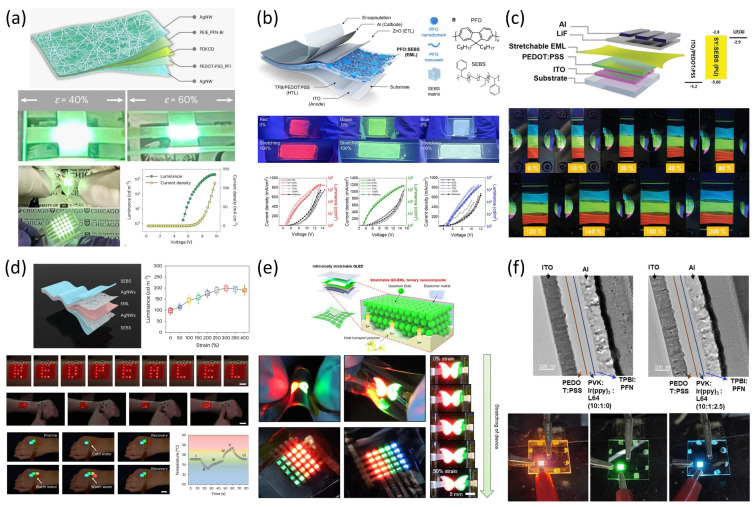
Stretchable Emissive Layers: representative device architectures and their behavior under mechanical deformation. (**a**) Fully stretchable OLEDs fabricated with PDKCD. Reprinted with permission from Ref. [12]. © 2024, Springer Nature. (**b**) Intrinsically stretchable blend films of organic light-emitting polymer formed nanodomains and a nanoweb network structure. Adapted from Ref. [13]. © 2023, The Authors. Published by Springer Nature under a Creative Commons CC BY license. (**c**) Images of films obtained from mixtures of TCTA:SEBS (blue film), green EML (green film), and red EML (red film) of an intrinsically stretchable emissive layer. Adapted with permission from Ref. [14]. © 2023, Wiley-VCH GmbH. (**d**) User-interactive skin display with a flexible PeACEL device. Adapted from Ref. [15]. © 2024, The Authors. Licensed under CC BY 4.0 (**e**) QD-based stretchable emission layer for intrinsically stretchable quantum dot light-emitting diodes. Adapted with permission from Ref. [16]. © 2024, Springer Nature. (**f**) Images of emitting PHOLEDs with isp-EML blended with different dopants (red, green, blue). Adapted with permission from Ref. [17]. © 2023, The Authors. Published by the American Chemical Society.

Early attempts focused on blending emissive polymers with elastomeric matrices to impart mechanical deformability while preserving electroluminescent properties. A notable study by Liu et al. introduced a thermally activated delayed fluorescence (TADF) polymer composite that achieved both high quantum efficiency and stretchability over 60%, marking a significant milestone in combining optical and mechanical performance [12] (Figure 2a). These materials are typically designed to retain phase stability under strain, which is critical for ensuring uniform light emission and preventing charge trapping.

Another important contribution came from Jeong et al., who developed intrinsically stretchable RGB emissive films by blending conjugated polymers with stretchable elastomers [13] (Figure 2b). This approach enabled full-color operation in deformable OLED arrays, demonstrating compatibility with large-area fabrication and multicolor emission control. Similarly, Xu et al. fabricated high-efficiency stretchable green and red phosphorescent OLEDs by engineering a soft matrix for the emissive layer that exhibited resilience under both uniaxial and biaxial strain [14] (Figure 2c). These studies collectively demonstrate that polymer chemistry, crosslinking strategies, and phase morphology optimization play central roles in determining stretchable EL layer performance.

Perovskite-based systems have also been adapted for stretchable display applications. In a recent breakthrough, Chun et al. reported multicolor perovskite electroluminescent devices integrated into skin-conformal platforms with up to 400% stretchability [15] (Figure 2d). Through careful structural engineering of the emissive layer and encapsulation, they achieved stable operation under dynamic motion, including bending, twisting, and stretching. The combination of quantum efficiency, spectral tunability, and mechanical robustness positions perovskites as promising candidates for future stretchable displays.

Quantum dot (QD)-based emissive materials offer additional advantages, including narrow emission bandwidth and high photostability. A study published in Nature Electronics detailed a fully stretchable QD-LED where the emissive layer was encapsulated within an elastomeric shell, maintaining emission intensity under >100% strain [16] (Figure 2e). This architecture allowed for excellent device retention across repeated deformation cycles and demonstrated scalability for wearable or skin-integrated displays.

Lastly, other studies have explored phosphorescent materials with inherent stretchability. For instance, a 2023 report in ACS Applied Materials & Interfaces showed how mechanically robust phosphorescent blends could be incorporated into epidermal displays without significant loss of luminance [17] (Figure 2f). By combining a soft matrix with highly emissive dopants and using strain-accommodating interfaces, these devices preserved color fidelity and quantum yield even under repetitive elongation.

Despite these advances, several technical hurdles persist. Most notably, the trade-off between mechanical compliance and charge carrier mobility remains a bottleneck. Highly stretchable polymers tend to exhibit lower crystallinity, which reduces charge injection and transport efficiency. Furthermore, maintaining uniform film thickness during stretching is challenging, often leading to light non-uniformity or local dielectric breakdown. To overcome these issues, future work will likely focus on novel copolymer designs, interpenetrating networks, or dynamic bonding chemistries that allow for energy dissipation and self-recovery under strain.

In conclusion, stretchable emissive layers have rapidly evolved from elastomeric blends to sophisticated composite systems capable of delivering high-efficiency electroluminescence under mechanical deformation. These developments serve as the cornerstone for next-generation stretchable displays with biomedical, wearable, and soft robotics applications.

#### 2.1.3. All-Intrinsic Devices (Fully Stretchable Stacks)

While considerable progress has been made in developing individual stretchable components such as electrodes or emissive layers, the realization of truly deformable display systems requires the seamless integration of all key layers—substrate, electrodes, emissive layers, encapsulation, and interconnects—into a single stretchable architecture. These fully intrinsic devices are designed to endure large, repeated mechanical deformations without delamination, cracking, or failure, while maintaining stable electrical and optical performance. Unlike hybrid approaches that combine stretchable and rigid materials, all-intrinsic systems aim to preserve uniform mechanical behavior across the entire device structure. Achieving this level of integration poses complex challenges due to the mechanical and chemical incompatibility between layers, as well as the trade-offs between stretchability, processability, and electronic functionality.

Recent comprehensive reviews, such as the work by Tran Quang Trung and Lee [18,19], highlight the inherent advantages of intrinsically stretchable components, including simplified fabrication processes, improved layer integrity, and cost-effectiveness compared to systems employing non-stretchable parts. Their review thoroughly covers the development of intrinsically stretchable conductors, semiconductors, and insulators, along with innovative fabrication methodologies enabling the construction of fully stretchable devices. Notably, their discussion encompasses recent advancements in stretchable field-effect transistors (FETs), photodetectors, light-emitting diodes (LEDs), electronic skins, and energy harvesters, emphasizing the technological innovations and challenges faced by the field.

Building on this foundation, composite device structures have been designed using chemically crosslinked networks that provide both electrical functionality and mechanical durability. In a 2023 study, a self-confined electroluminescent elastomer was reported that exhibited high efficiency and reliability in stretchable OLEDs without requiring traditional blending or multilayer stacking [20]. The material architecture leveraged dynamic bonding to localize emission and reduce interfacial delamination, enabling up to 50% stretchability while preserving light output uniformity. This method simplified the device structure while improving energy transfer efficiency and operational lifetime under strain, illustrating a promising route for minimizing complexity in fully stretchable stacks.

Another innovative example comes from Li et al., who developed a soft “drawing display” system based on conductive inks and light-emitting materials that could be painted, peeled, and reused on various stretchable substrates [21]. The entire system—emissive layers, electrodes, and dielectric interfaces—was fully compliant with strain, and the printed patterns retained functional performance even after being stretched, twisted, or reshaped. This technique represents a new paradigm in personalized and reconfigurable electronics, enabling low-cost and rapid prototyping of stretchable displays for education, interactive signage, and on-skin visual interfaces.

In a notable demonstration of fully intrinsic OLEDs, Kim and Park reported a device in which all active layers—including the electrodes, charge transport layers, and emissive layer—were constructed from deformable materials [22]. Their integrated system maintained stable electroluminescence under up to 80% uniaxial strain and showed minimal performance degradation after 200 cycles. Interestingly, the device exhibited enhanced emission under moderate strain due to strain-aligned polymer domains, underscoring the importance of mechanical-electrical coupling in stretchable displays. The study also emphasized the benefits of using fully polymeric systems with matched elasticity between layers, which reduces stress concentration and delays fatigue-driven failure, a common limitation in many previous designs.

Despite these advancements, fully integrated stretchable display systems still face significant bottlenecks. One major issue is cumulative mechanical fatigue, which often results in delamination at multilayer interfaces or degradation of light-emitting efficiency. Furthermore, aligning fabrication processes for each stretchable component—particularly over large-area substrates—is still technically demanding. Even small mismatches in thermal or mechanical properties between layers can lead to warping or performance drift over time. To overcome these limitations, future strategies may include the use of self-healing materials, molecularly entangled interfacial layers, or additive manufacturing techniques such as multi-material 3D printing to co-fabricate all device layers with tailored mechanical gradients. Such approaches could enable not only greater durability but also device miniaturization and design freedom for next-generation applications.

### 2.2. Wavy Surface Displays

Wavy surface displays achieve mechanical stretchability not through the use of intrinsically deformable materials, but via geometric engineering that introduces out-of-plane deformation into otherwise rigid layers. By leveraging structural features such as buckles and wrinkles, these approaches enable stretchable behavior in displays built from conventional high-performance electronic materials. The resulting “wavy” geometries act as mechanical buffers, absorbing strain by geometric displacement rather than material extension, thereby maintaining device functionality under repeated deformation.

This class of design is particularly attractive for hybrid systems, where rigid components—like inorganic LEDs or TFTs—must operate within a compliant, wearable form factor. Wavy architectures offer high strain tolerance, minimal interference with electrical/optical performance, and are often compatible with scalable manufacturing methods. Depending on how the out-of-plane deformation is introduced, two primary strategies are distinguished: buckled structures, which arise from prestrain-release mechanics, and wrinkled surfaces, which emerge from spontaneous instability due to modulus mismatch.

In the following subsections, both strategies are discussed in detail, with an emphasis on their formation mechanisms, mechanical behavior, and practical applications in stretchable display systems.

#### 2.2.1. Buckled Structures

Buckling-based architectures represent one of the most effective and widely adopted structural strategies for achieving mechanical stretchability in display systems. Rather than relying on intrinsically deformable materials, buckled structures enable the incorporation of rigid or brittle functional components—such as inorganic light-emitting diodes (LEDs), thin-film transistors (TFTs), and photodetectors—into soft, elastic substrates.

Buckling refers to a form of mechanical instability in which thin films undergo out-of-plane deformation, such as wave-like undulations, in response to compressive stress. This physical phenomenon is harnessed in stretchable electronics to form predesigned geometries that buffer strain and prevent material failure, enabling stretchability without altering the intrinsic properties of rigid functional layers. This is accomplished through intentional out-of-plane deformation generated by mechanical instability, typically in the form of wave-like geometries that buffer strain during stretching. The resulting systems retain the performance advantages of high-mobility inorganic materials while attaining substantial mechanical compliance.

The fundamental concept of buckling-based design involves bonding thin functional films onto pre-stretched elastomeric substrates. Upon release of the pre-strain, compressive stress induces the spontaneous formation of periodic buckles, typically sinusoidal or mesh-like in shape, depending on the layout and boundary conditions of the system. These buckled geometries act as strain reservoirs, allowing the entire structure to deform by unfolding rather than stretching the rigid materials themselves.

**Figure 3:**
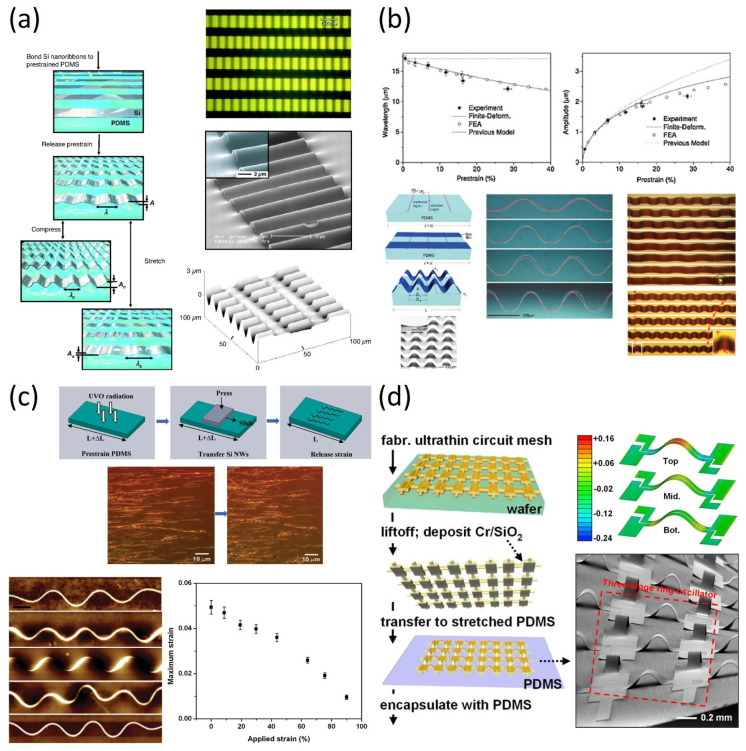
Buckled structures for stretchable electronic systems. (**a**) Schematic illustration of the fabrication process for wavy, single-crystal Si ribbons on pre-strained PDMS substrates, along with optical, SEM, and AFM images of the resulting buckled geometries. Adapted from Ref. [23]. Copyright 2007, National Academy of Sciences. (**b**) Wavelength and amplitude of buckled Si ribbons as a function of applied prestrain, along with schematic of the controlled delamination buckling process. Adapted with permission from Ref. [24]. © 2009, Wiley-VCH Verlag GmbH & Co. KGaA, Weinheim. (**c**) Fabrication of 3D-buckled silicon nanowires using UVO-treated substrates, with large-area optical and AFM images, and calculated maximum strain of oval-coiled nanowire structures under tensile deformation. Adapted with permission from Ref. [26]. © 2011, American Chemical Society. (**d**) Formation of stretchable electrodes via buckled interconnects, including optical images, FEM simulation results, and device-level illustration of ultrathin circuit mesh transferred onto pre-strained PDMS. Adapted from Ref. [27]. Copyright 2008, National Academy of Sciences.

A pivotal work by Jiang et al. advanced the theoretical understanding of these systems by applying finite deformation mechanics to describe how thin films buckle on compliant substrates [23], as shown in Figure 3a. Their analysis provided a predictive framework for determining the wavelength, amplitude, and strain distribution of buckled structures, guiding the design of systems with tunable mechanical responses.

Building on this foundation, buckling strategies have been successfully implemented in stretchable electronics. Khang et al. demonstrated that mechanically induced buckling of thin film devices on pre-strained elastomeric substrates can create wavy structures that accommodate large deformation without failure [24] (Figure 3b). These principles have been extended to stretchable display applications, where buckled interconnect geometries help maintain electrical performance under strain. This approach is particularly suitable for active matrix integration, enabling both mechanical robustness and electrical continuity.

More sophisticated implementations of buckling have emerged, involving hierarchical or multiscale architectures. Sun et al. reported the controlled buckling of semiconductor nanoribbons to produce stretchable field-effect transistors and circuits with high areal coverage [25]. Their method allowed for precise spatial modulation of buckling parameters, enabling device-level control over mechanical behavior without compromising electrical performance. These results demonstrated that buckled structures can be predictably designed and integrated into multilayer functional systems.

Expanding on this concept, Xu et al. introduced a 3D buckling technique for silicon nanowires, achieving controlled assembly of complex out-of-plane architectures [26], illustrated in Figure 3c. This three-dimensional approach offered enhanced stretchability and allowed for the integration of vertical functional features, paving the way for new classes of bio-integrated and skin-conformal electronics.

Despite the significant mechanical advantages of buckled structures, challenges remain in achieving high-resolution pixel integration and multilayer alignment over large areas. The inherent surface topography introduced by buckling can interfere with layer stacking, and precision in strain programming is required for reproducible results. Furthermore, long-term fatigue behavior and delamination resistance must be carefully addressed in display environments involving repeated bending and stretching.

A notable variation on this concept was demonstrated by Kim et al., who implemented noncoplanar mesh designs in stretchable circuits using ultrathin buckled interconnects embedded within soft substrates [27] (Figure 3d). Although often associated with serpentine geometries, their architecture also relied on controlled out-of-plane deformation of the electrode network, providing a mechanically compliant interconnect layout that preserved electrical continuity under extreme deformation. This approach highlights the versatility of buckling principles beyond traditional sinusoidal forms and their applicability to mesh-patterned electronics.

In conclusion, buckled structures provide a powerful strategy for mechanically isolating rigid electronic components from external strain through geometric design. Their tunability, compatibility with high-performance materials, and demonstrated success in stretchable displays make them a cornerstone of current structural approaches. Future efforts may focus on scalable fabrication methods, such as transfer printing and laser patterning, as well as dynamic reconfiguration through stimuli-responsive substrates to further advance the capabilities of buckled stretchable electronics.

#### 2.2.2. Wrinkled Surfaces

Wrinkled surface architectures represent a spontaneous and material-driven strategy for achieving mechanical compliance in stretchable display systems. Unlike buckled structures that rely on deliberate mechanical pre-straining and geometric design, wrinkles arise from surface instabilities triggered by intrinsic mismatches in mechanical properties—such as elastic modulus and thermal expansion—between adjacent layers. These instabilities induce self-organized, periodic surface topographies that act as stress-relief reservoirs, enabling rigid thin films to withstand deformation without cracking or delamination.

Wrinkle formation is typically induced by depositing a thin, stiff functional layer—such as a metal, oxide, or emissive organic material—onto a soft elastomeric substrate that has been pre-stretched or subjected to a thermal gradient. Upon release of the strain or thermal equilibration, compressive stress builds at the interface, leading to sinusoidal or hierarchical wrinkle patterns. The wavelength and amplitude of these wrinkles depend on parameters such as film thickness, mechanical stiffness ratio, and the degree of prestrain, allowing fine control over the final morphology for target device applications.

**Figure 4:**
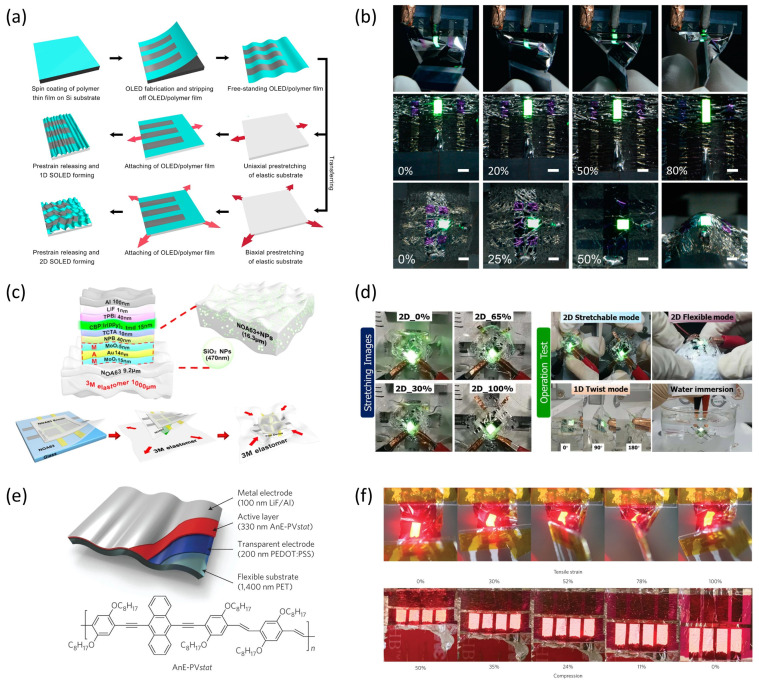
Wrinkled structure-enabled stretchable light-emitting devices. (**a**) Schematic of the fabrication process of 1D and 2D stretchable OLEDs (SOLEDs) via prestraining and transfer printing of ultrathin OLED/polymer films. (**b**) Demonstration of the ultraflexibility of a free-standing ultrathin OLED under buckling and twisting. Adapted with permission from Ref. [28]. © 2016, American Chemical Society. (**c**) Schematic illustration of the layer structure and fabrication of GSOLEDs, highlighting the device composition without nanoparticle integration. (**d**) Optical performance of GSOLEDs under various two-dimensional stretching conditions (0–100%), including operation tests under 2D stretchable, 2D flexible, 1D twisting, and water immersion conditions. NOA63 encapsulation enables environmental stability in liquid media. Adapted from Ref. [29]. © 2021, The Authors. Licensed under CC BY 4.0 (**e**) Cross-sectional schematic of multilayer PLEDs with realistic thicknesses and material composition, alongside the chemical structure of the emissive polymer AnE-PVstat. (**f**) Photographic demonstrations of ultrathin PLEDs under extreme mechanical deformation, including bending, stretching, and compression. Adapted with permission from Ref. [30]. © 2013, Springer Nature.

In display technologies, wrinkled surfaces have been successfully applied to stretchable light-emitting diodes, including OLEDs and polymer LEDs (PLEDs), to enhance both stretchability and optical performance. For instance, Yin et al. demonstrated a two-dimensionally stretchable OLED system fabricated via controlled wrinkling of ultrathin emissive layers, achieving up to 50% areal strain while maintaining a high efficiency of 79 cd/A [28] (Figure 4a,b). These wrinkled devices exhibited robust electroluminescence with minimal performance degradation under repeated stretching cycles.

Choi et al. introduced a high-performance wrinkled OLED using a MoO_3_/Au/MoO_3_ transparent electrode architecture that enabled superior heat dissipation and out-coupling efficiency [29] (Figure 4c,d). The wrinkled surface not only preserved emission uniformity under 2D random strain but also enhanced device lifetime through thermal management.

Meanwhile, White et al. reported ultrathin and stretch-compatible PLEDs that could endure up to 100% strain, attributing their mechanical durability and stable performance to the intrinsic flexibility of the polymer materials and the stress-dissipating nature of the wrinkled structure [30] (Figure 4e,f).

The advantages of wrinkled surfaces extend beyond mechanical resilience. These structures can manipulate light propagation and scattering, reducing reflectivity and broadening the viewing angle—key attributes for wearable displays and conformal lighting systems. Additionally, since wrinkle formation is based on self-assembly, it is inherently scalable and compatible with roll-to-roll manufacturing, making it suitable for large-area and low-cost device production.

Despite these benefits, challenges remain in achieving wrinkle uniformity over curved or multilayered substrates and ensuring long-term stability under environmental stressors. Continued advances in strain-guided alignment, adhesive interlayer design, and responsive materials are expected to further expand the applicability of wrinkled architectures in future stretchable display platforms.

### 2.3. Hybrid Stretchable Displays

Hybrid stretchable display architectures represent a practical and scalable solution to the challenges of integrating high-performance electronic components into deformable systems. While intrinsically stretchable materials offer attractive mechanical properties, they often lag behind in terms of electrical performance, process compatibility, or long-term reliability. Hybrid approaches aim to circumvent these limitations by combining conventional, rigid microelectronic elements—such as micro-LEDs, thin-film transistors (TFTs), or quantum dot emitters—with soft, elastic substrates through structural engineering and layout optimization. This enables the development of display systems that are not only mechanically stretchable but also capable of delivering high-resolution, brightness, and color purity comparable to their rigid counterparts.

Central to this strategy is the decoupling of mechanical stress from active functional regions, allowing rigid islands or interconnects to remain electrically stable under deformation. Rather than relying solely on new materials, hybrid designs exploit geometrical motifs and spatial configurations to localize and redistribute strain across the device. This design freedom facilitates the use of existing semiconductor technologies, which is particularly advantageous for industrial scalability and system-level integration.

In this section, two representative and complementary structural strategies are discussed: serpentine wiring and island–bridge configurations. Serpentine wiring focuses on the design of electrical interconnections by patterning metallic conductors in wavy or meandering forms. These geometries absorb mechanical deformation geometrically, allowing the conductors to maintain electrical integrity even under high strain. In contrast, island–bridge configurations emphasize the global layout of the device. Rigid functional components (“islands”) are spatially distributed on a soft substrate and connected by highly stretchable bridges. This arrangement ensures that sensitive electronic regions remain largely unaffected by mechanical strain, as the deformation is absorbed primarily by the bridge regions.

The following subsections review these strategies in detail, including their mechanical principles, design considerations, fabrication techniques, and representative research outcomes. Together, these approaches form the backbone of hybrid stretchable display systems that enable both mechanical adaptability and high electronic performance.

#### 2.3.1. Serpentine Wiring

**Figure 5:**
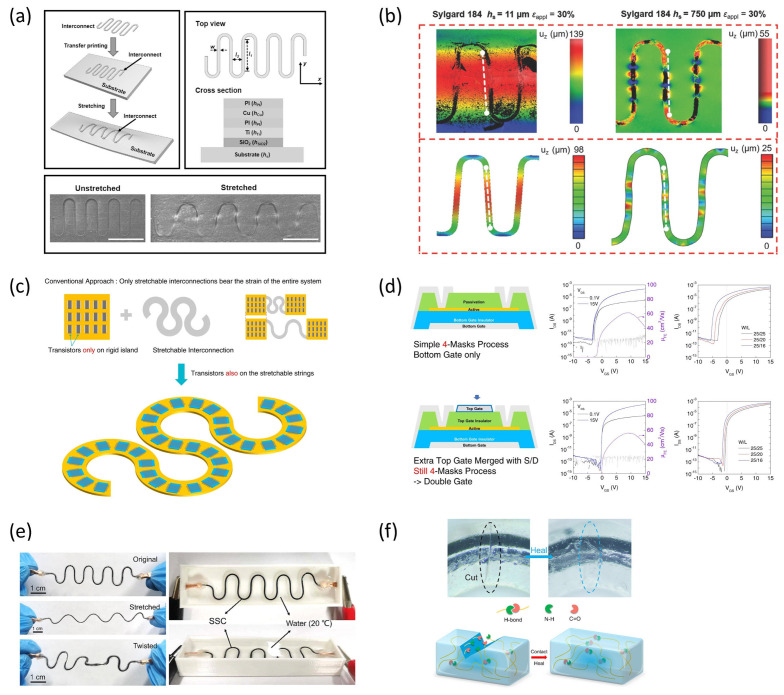
Representative Demonstrations of Serpentine Wiring Strategies in Stretchable Electronics. (**a**) Schematic illustration of the fabrication process for planar serpentine interconnects on soft elastomeric substrates via transfer printing, along with optical images comparing the morphology of interconnects in unstretched and stretched states. (**b**) Experimental 3D optical profilometry and corresponding finite element analysis (FEA) results showing out-of-plane displacement distributions of serpentine interconnects fabricated on Sylgard 184 substrates with different thicknesses under 30% tensile strain. Adapted with permission from Ref. [33]. © 2017, Wiley-VCH Verlag GmbH & Co. KGaA, Weinheim. (**c**) Design concept of high-density stretchable transistor arrays where inorganic TFTs are embedded directly on serpentine wiring structures to achieve full mechanical compliance. (**d**) Fabricated oxide thin-film transistors (TFTs) based on enhanced multi-gate structures, enabling stretchable device operation with improved electrical performance and simplified lithographic processing. Adapted from Ref. [34]. © 2022, The Authors. Licensed under CC BY 4.0 (**e**) Photographic demonstrations of a self-healing serpentine conductor (SSC) under stretching, twisting, and immersion in water, illustrating its robustness and waterproof characteristics. (**f**) Optical images of the SSC surface before and after self-healing, and schematic representation of the self-healing mechanism based on hydrogen bonding and reversible dynamic interactions within the WPU sheath. Adapted with permission from Ref. [35]. © 2023, Elsevier.

Serpentine wiring is one of the most widely adopted and rigorously investigated strategies in hybrid stretchable displays, offering a geometrically engineered solution to maintain electrical continuity under mechanical deformation. Unlike intrinsically stretchable conductors, which often suffer from conductivity degradation under strain, serpentine-shaped interconnects deform by unfolding their meandering geometry, effectively redistributing strain and minimizing local stress concentration [31]. The fundamental design involves embedding or patterning ductile metals—such as gold, copper, or silver—into sinusoidal or spiral configurations on soft elastomeric substrates. Under stretching, these interconnects accommodate strain not by elongating the metal itself but by geometric reconfiguration, including in-plane elongation and out-of-plane buckling. This design principle allows them to sustain tensile strains exceeding 100% without electrical or structural failure [32,33]. Advanced modeling using finite deformation theory, such as by Fan et al., has refined our understanding of these behaviors beyond the limitations of infinitesimal strain models [32]. Pan et al. further demonstrated that thinner elastomer substrates induce global buckling of serpentine structures, enabling higher elastic stretchability before plastic deformation sets in (Figure 5a,b) [33].

This strategy has been widely adopted in the development of high-performance stretchable displays. For example, Oh et al. demonstrated a highly integrated stretchable transistor array by embedding oxide thin-film transistors into serpentine interconnects, thereby overcoming the traditional trade-off between stretchability and integration density. The resulting system maintained excellent electrical performance under tensile stress, facilitated by a multi-gate structure and simplified mask process (Figure 5c,d) [34]. Additionally, innovative materials have been developed to enhance mechanical robustness. Yuan et al. introduced a sheath-core structured conductive yarn that remains electrically stable even under 100% strain and offers excellent waterproofing and self-healing properties, expanding the feasibility of serpentine wiring in harsh environments (Figure 5e,f) [35].

While serpentine interconnects have demonstrated excellent stretchability by distributing mechanical strain over curved paths, their long-term mechanical reliability remains a concern, particularly at junction points, where stress concentration can lead to fatigue failure under cyclic deformation. In practical applications, interconnects must endure repeated stretching and relaxation without developing cracks or delamination. Comparative studies have evaluated a range of interconnect geometries, such as horseshoe, fractal-inspired, and mesh-based designs, which aim to balance mechanical robustness with footprint efficiency.

For instance, Fan et al. developed a finite deformation model of planar serpentine interconnects and quantitatively analyzed the inherent trade-offs between footprint area and stretchability across various geometries [32]. In parallel, Xu et al. demonstrated that fractal geometries can significantly improve fatigue resistance by enabling hierarchical strain dispersion and reducing stress concentration [36]. A quantitative understanding of these geometry-dependent behaviors is crucial for optimizing wiring design in high-performance stretchable display systems.

Meanwhile, Koshi et al. examined the fatigue lifetime of serpentine copper interconnects under repeated mechanical stress and washing. They found that laminated structures and applied strain amplitudes critically influence cycle durability, with optimized structures surviving multiple washing cycles while retaining electrical function in textile-based temperature monitoring systems [37]. The applicability of serpentine wiring is further highlighted in epidermal systems that incorporate serpentine-patterned circuits into deformable platforms, demonstrating seamless integration for physiological signal acquisition, processing, and storage using carbon nanotube-based flash memory arrays [38].

In summary, serpentine wiring exemplifies a structurally driven approach that combines material science, geometric design, and system-level integration. Its continued refinement is key to achieving highly stretchable, reliable, and high-resolution display platforms for wearable, implantable, and conformal electronic applications.

#### 2.3.2. Island–Bridge Configurations

The island–bridge configuration is a prominent and widely adopted strategy for achieving hybrid stretchability in display systems. In this design, rigid or semi-rigid functional units—referred to as “islands”—are distributed across a deformable substrate and electrically interconnected via highly stretchable “bridges.” This architectural approach enables the integration of high-performance yet brittle components, such as micro-LEDs, inorganic thin-film transistors (TFTs), or quantum dot (QD) emitters, into systems that require mechanical compliance. While serpentine wiring primarily focuses on stress absorption through interconnect geometry, the island–bridge design emphasizes system-level strain isolation by physically decoupling deformation zones from active electronic regions [39].

In typical implementations, islands are composed of microelectronic components pre-fabricated on rigid or semi-rigid platforms. These islands are then spatially arranged onto an elastomeric matrix, often using transfer printing or pick-and-place methods. The stretchable bridges connecting these islands may take the form of serpentine metal wires, liquid metal traces, or nanomesh conductors. During mechanical stretching, deformation is absorbed by the bridges, allowing the islands to remain mechanically and electrically stable. This design allows for excellent preservation of optical and electronic performance even under large and repeated strain cycles [40].

**Figure 6:**
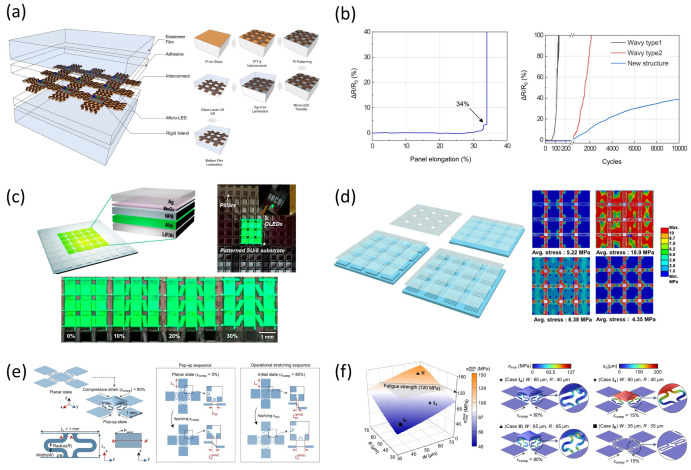
Schematic and performance analysis of stretchable display systems based on island–bridge configurations. (**a**) Fabrication process and cross-sectional structure of a stretchable display incorporating rigid micro-LED islands interconnected via deformable bridges. (**b**) Operational characteristics of the stretchable panel under biaxial elongation, demonstrating mechanical durability up to 34% strain. Adapted with permission from Ref. [41]. © 2023, Society for Information Display. (**c**) Stretchable OLED architecture incorporating vertically aligned stress-relief pillars between active layers and substrate for enhanced mechanical compliance. (**d**) Finite element simulation showing reduced stress concentration in the pillar-supported OLED structure. Adapted with permission from Ref. [42]. © 2020, American Chemical Society. (**e**) Optical and 3D deformation profiles of the stretchable OLED under various strain levels. (**f**) FEA results illustrating strain distribution and vertical displacement in pillar-integrated OLED structures under mechanical stretching. Adapted from Ref. [43]. © 2024, The Authors. Licensed under CC BY 4.0.

Jung et al. exemplified this concept with a high-resolution active-matrix micro-LED display, wherein rigid μLED chips were mounted on soft substrates and connected by serpentine interconnects (Figure 6a,b) [41]. Their display achieved uniform luminance and operational stability under >20% biaxial strain, showcasing the robust mechanical-electrical decoupling enabled by this approach. The use of transfer printing to assemble micro-LEDs at precise locations demonstrated its compatibility with high-density device layouts and scalable fabrication processes.

In addition to micro-LEDs, island–bridge strategies have been extended to organic light-emitting devices. Lim et al. developed a stretchable OLED using vertically aligned micropillar arrays to buffer mechanical stress between rigid emitter layers and the soft substrate (Figure 6c,d) [42]. This vertical decoupling method proved effective in preserving device luminance (retaining >80% brightness under 30% strain), illustrating the importance of three-dimensional mechanical design in hybrid stretchable electronics.

Further innovation was demonstrated by Kim et al., who proposed 3D height-alternant island arrays for OLED systems (Figure 6e,f) [43]. This approach not only improved strain distribution across the substrate but also significantly increased the active area ratio—a key metric in achieving visually immersive displays.

Similarly, Park et al. utilized a facile photo-patterning technique to fabricate stretchable OLEDs with integrated bridges, achieving fine resolution and mechanical durability suitable for scalable production lines [44]. Beyond conventional layouts, shape-adaptive systems have also emerged. Rao et al. introduced deformable micro-LED structures interconnected by highly elastic bridges, enabling conformable, skin-like displays suitable for non-planar and dynamic surfaces such as human skin or soft robotic actuators [45]. This study highlights the potential of island–bridge configurations in biomedical and wearable applications, where both stretchability and intimate skin contact are critical.

Design optimization for island–bridge systems involves fine-tuning multiple parameters: the size and density of islands, the mechanical modulus and geometry of bridges, and the encapsulation approach. Larger spacing between islands increases stretchability but may reduce resolution, necessitating trade-offs based on target application. Finite element analysis (FEA) is often used to simulate strain fields and guide design iterations, particularly around interconnect junctions where stress concentration is likely.

Despite these achievements, several technical hurdles remain, including ensuring strong adhesion between heterogeneous materials, preventing fatigue failure under repeated deformation, and aligning multilayer device stacks with high precision. Among these challenges, interfacial stability between rigid and soft materials is often emphasized, particularly in discussions of delamination and thermal mismatch. While these issues can arise at the interface between rigid islands and stretchable substrates, they are often effectively addressed in practical systems. In particular, when using elastomeric substrates such as PDMS or other silicone-based stretchable films, the incorporation of appropriately selected silicone-based or acrylic-based adhesive layers can relieve interfacial stress and ensure mechanical stability. As such, while these interfacial concerns warrant careful consideration, they are generally well-managed through appropriate material and process selection, and thus do not fundamentally limit the advancement of island–bridge systems.

Building on these insights, a comparative summary of the three primary strategies—intrinsic, wavy, and hybrid—is provided in Table 1. This table outlines the mechanical design principles, commonly used materials, stretchability ranges, and characteristic trade-offs for each approach.

## 3. Applications and Future Directions

As stretchable display technologies mature beyond laboratory prototypes, their integration into real-world systems has become increasingly feasible. Diverse application domains are beginning to adopt stretchable visual interfaces that offer not only mechanical adaptability but also functional innovation. This section highlights representative application areas where stretchable displays have demonstrated significant impact or future potential. Furthermore, we outline the technological directions required to overcome remaining barriers and advance these systems toward widespread deployment.

### 3.1. Applications

The rapid advancement of stretchable display technologies has unlocked a broad spectrum of applications that capitalize on their unique mechanical compliance and optical functionalities. These applications span from intimate healthcare monitoring systems to interactive textiles, implantable biomedical devices, and responsive soft robotics, all of which benefit from displays capable of enduring complex deformations without performance loss.

In wearable healthcare and electronic skin, stretchable displays provide real-time physiological feedback and interface capabilities directly on the body, enhancing personalized medicine and patient monitoring. Smart textiles leverage the integration of stretchable displays and sensors into fabric structures, enabling dynamic fashion and multifunctional garments. Implantable biomedical devices utilize biocompatible stretchable displays and electronic components to achieve minimally invasive, real-time diagnostics and therapeutics within the body. Meanwhile, soft robotics and human–machine interfaces incorporate stretchable displays and sensors to create adaptable, responsive systems that interact seamlessly with humans and complex environments.

This section comprehensively explores these diverse application domains, highlighting recent technological breakthroughs, ongoing challenges, and future prospects in leveraging stretchable display technologies to redefine human-device interaction across multiple fields.

#### 3.1.1. Wearable Healthcare and Electronic Skin

Stretchable displays have emerged as a transformative technology in wearable healthcare systems and electronic skin (e-skin) platforms, offering unprecedented opportunities for continuous health monitoring and seamless human-machine interaction. These systems demand intimate and conformal contact with the human body, operating on soft, curved, and dynamically deforming surfaces such as skin, joints, and muscles. Unlike rigid or merely flexible displays, stretchable displays can accommodate multi-axial strains without compromising optical or electrical performance, making them uniquely suited for on-skin applications.

**Figure 7:**
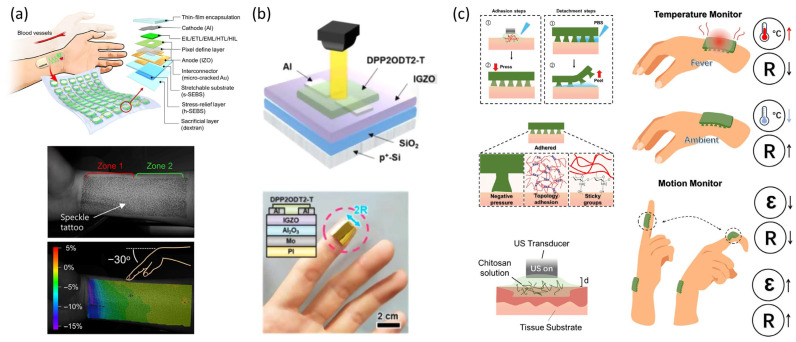
Applications of electronic skin for wearable healthcare and physiological monitoring. (**a**) Structural schematic and implementation of a standalone stretchable organic optoelectronic health patch (SHP) for real-time heart rate monitoring using integrated OLEDs and photodiodes. Adapted from Ref. [46]. © 2021, The Authors. Licensed under CC BY-NC 4.0 (**b**) Structure and operation of skin-conformable photoplethysmogram (PPG) sensors using low-power oxide semiconductors; schematic of the dual-mode operation for continuous cardiovascular monitoring. Adapted with permission from Ref. [47]. © 2022, Elsevier. (**c**) Schematic of adhesion/detachment mechanism of hydrogel-based patches with negative pressure and chemical interactions, and illustration of temperature and motion monitoring using bioinspired wearable interfaces. Adapted with permission from Ref. [49]. © 2024, Acta Materialia Inc. Published by Elsevier Ltd.

One notable advancement in this domain is the development of standalone organic stretchable health patches (SHPs) that integrate stretchable organic light-emitting diode (OLED) displays with photodiodes. For example, a 15-μm-thick device was demonstrated to conform closely to the skin surface, enabling real-time heart rate monitoring without the need for bulky external equipment [46] (Figure 7a). This integration facilitates continuous physiological feedback, enhances wearer comfort, and paves the way for personalized healthcare devices that operate unobtrusively throughout daily activities.

In parallel, skin-conformable photoplethysmogram (PPG) sensors leveraging low-power oxide semiconductors have been developed to achieve energy-efficient, always-on cardiovascular monitoring. These devices utilize flexible indium gallium zinc oxide (IGZO) phototransistors paired with miniaturized LEDs, providing high sensitivity and reliability with minimal power consumption [47] (Figure 7b). The coexistence of these two approaches—display-integrated patches and ultra-low-power sensing—demonstrates the spectrum of strategies in wearable healthcare, balancing functional complexity and energy constraints.

Beyond these, recent research has significantly advanced 3D-printed electronic skin platforms embedding multifunctional sensors capable of detecting strain, pressure, and temperature. While these platforms do not yet incorporate emissive display components, their mechanically robust, highly conformable architectures provide a promising substrate for future hybrid e-skin devices that combine sensing and display functionalities, fostering fully interactive and intelligent skin-like interfaces [48].

Furthermore, bioinspired skin–electronic interfaces have been engineered with enhanced adhesion and conformal contact, fabricated through innovative multi-coupled 3D printing techniques. These interfaces maintain stable operation during dynamic body movements and long-term wear, making them particularly suitable for continuous physiological monitoring and therapeutic applications. They also present a viable platform for integrating stretchable optoelectronic modules in future wearable devices, facilitating durable and user-friendly biomedical electronics [49] (Figure 7c).

Collectively, these advancements not only expand the functional capabilities of wearable healthcare technologies but also address critical challenges such as mechanical compliance, biocompatibility, power efficiency, and durability. Looking forward, the integration of stretchable displays with multifunctional sensors and intelligent data analytics promises to revolutionize personalized medicine, enabling proactive health management and real-time disease prevention through sophisticated, skin-like wearable systems.

#### 3.1.2. Smart Textiles and Fashion Integration

Smart textiles represent an emerging frontier in wearable technology, where electronic functionalities are seamlessly integrated into traditional fabrics to create garments that are flexible, comfortable, and interactive. At the core of this innovation lies the development of smart fibers and textiles, which are highly desirable due to their lightweight, breathability, weavability, stretchability, and exceptional adaptability to diverse mechanical deformations during everyday human activity. These features enable textiles not only to serve as passive clothing materials but also as active platforms for display, sensing, and communication. Hou et al. [50] provide a comprehensive overview of fiber- and textile-based breathable substrates, conductive materials, and their diverse roles in wearable electronics, such as stretchable conductive fibers, soft sensors, and personal healthcare devices. The review further discusses scalable manufacturing methods and functional design strategies that prioritize comfort and mechanical robustness, offering valuable insights for the advancement of clothing-based wearable systems.

**Figure 8:**
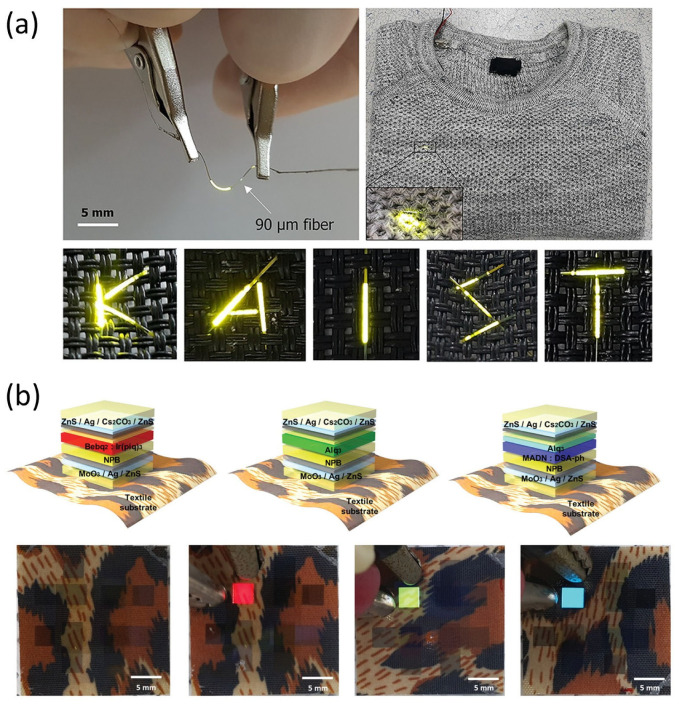
Smart textile implementations based on stretchable display technology. (**a**) Weavable and highly efficient organic light-emitting fibers integrated into textile substrates, enabling flexible and durable emission across complex surfaces. Adapted with permission from Ref. [51]. © 2018, American Chemical Society. (**b**) RGB-color textile-based flexible and transparent OLEDs demonstrating high-quality color rendering and seamless textile compatibility for aesthetic wearable applications. Reproduced from Ref. [52]. © 2022, The Authors. Licensed under CC BY 4.0.

One of the most promising technological breakthroughs within this domain is the fabrication of organic light-emitting fibers (OLEFs) that can be woven directly into fabric structures. Kwon et al. [51] demonstrated a highly efficient and scalable low-temperature manufacturing process for OLEFs, which are capable of maintaining excellent optical and mechanical performance even when subjected to repetitive bending and stretching. These weavable OLED fibers not only preserve the softness and breathability of textiles but also enable them to function as lightweight, flexible display surfaces. This achievement significantly enhances the practicality and functionality of smart clothing, bringing dynamic, emissive visual interfaces into everyday garments (Figure 8a).

Complementing this progress, Lee et al. [52] explored RGB-color textile-based flexible and transparent OLEDs, with a particular emphasis on their aesthetic appeal and textile compatibility. The study highlights devices that offer vibrant, high-quality color expression while maintaining transparency and flexibility, allowing seamless integration into fabric layers without compromising wearer comfort (Figure 8b). Such innovations underscore the potential for stretchable display technologies to bridge the gap between engineering functionality and fashion aesthetics, empowering designers to create garments that are both expressive and interactive.

The convergence of electronics and fashion opens new possibilities for personalized and adaptive aesthetics in clothing. Wearers can dynamically change colors, patterns, or visual content on their garments in response to environmental stimuli, physiological signals, or social contexts. Stoppa and Chiolerio [53] emphasized in their critical review the paradigm-shifting role of wearable electronics in the fashion industry, enabling garments that communicate identity, mood, or intention through responsive visual feedback. This evolution redefines garments not just as wearable tools but as communicative extensions of the human body.

Beyond aesthetic innovation, smart textiles offer tangible benefits in critical application areas such as healthcare, athletics, and defense. Integrated sensors and stretchable displays enable real-time physiological monitoring, gesture recognition, and environmental data collection, making garments multifunctional and interactive. However, several technical hurdles must be addressed to achieve commercial viability. Cherenack and Van Pieterson [54] analyzed key challenges, including mechanical durability under repeated strain, stable electrical interconnections under motion, washability, energy autonomy, and overall user comfort. Recent advancements in conductive yarns, robust encapsulation strategies, and textile-compatible energy harvesting systems are steadily overcoming these limitations, thereby accelerating the transition of smart textiles from laboratory prototypes to market-ready solutions.

Looking ahead, the synergy between textile engineering and stretchable display technologies is poised to revolutionize human–clothing interaction. Future smart garments are expected not only to deliver dynamic visual feedback and contextual information but also to respond intelligently to environmental changes or user intentions through embedded sensors, actuators, and AI-powered processing units. These next-generation wearable systems envision clothing as an integrated platform for communication, health monitoring, ambient awareness, and even emotional expression. As the field continues to evolve, smart textiles are set to redefine both the function and identity of fashion, creating a harmonious convergence of utility, beauty, and intelligence.

#### 3.1.3. Soft Robotics and Human–Machine Interfaces

Soft robotics represents a paradigm shift in robotics, emphasizing the use of compliant, deformable materials to create machines that can safely interact with humans and adapt to complex environments. The foundational review by Rus and Tolley [55] comprehensively discusses the design, fabrication, and control of soft robots, highlighting the importance of material stretchability and flexibility for enabling new functionalities beyond the reach of rigid systems.

Integrating stretchable electronic devices, including displays and sensors, into soft robotic platforms enhances their interactivity and adaptability. Stretchable displays can be conformally mounted on soft robotic surfaces, enabling real-time visual feedback and communication of the robot’s state or environment. Meanwhile, stretchable sensors provide vital inputs for closed-loop control, allowing robots to respond dynamically to physical stimuli and user commands.

Human–machine interfaces (HMIs) based on stretchable electronics extend these capabilities by creating skin-like interfaces that translate human motion, touch, and physiological signals into electronic commands. These advanced HMIs improve user experience and enable more natural and intuitive control over robotic or prosthetic devices, as well as augmented reality applications.

Pioneering studies in plastic bioelectronics have demonstrated the potential of flexible, stretchable materials to serve as electronic skins, wearable sensors, and soft actuators. Someya et al. [56] review recent progress in plastic bioelectronics, including organic electronic materials and devices that combine biocompatibility with mechanical compliance, paving the way for sophisticated soft robotics and human–machine interfaces.

In addition, stretchable carbon nanotube-based strain sensors have been developed for precise human-motion detection, providing robust and sensitive electronic skin functions crucial for soft robotic control and interactive HMIs [57]. Despite significant advances, challenges remain in enhancing device robustness, power efficiency, and integration density. Future research aims to develop multifunctional, self-powered, and miniaturized stretchable devices capable of seamless integration into soft robotic and HMI systems.

### 3.2. Future Directions

As stretchable display technology matures, future research is poised to tackle several critical challenges to realize its full potential across diverse applications. These challenges span material innovation, device architecture, system integration, and operational sustainability. To this end, four key directions emerge as focal points for advancing the field: achieving high-resolution stretchable displays, developing energy-autonomous systems, engineering durable encapsulation techniques, and integrating artificial intelligence for enhanced functionality.

These directions collectively aim to overcome current limitations by improving display quality and mechanical robustness, enabling self-sufficient power solutions, ensuring long-term device stability, and embedding intelligent capabilities. The following sections delve into each of these areas, highlighting recent progress, ongoing challenges, and promising avenues for future breakthroughs.

#### 3.2.1. High-Resolution Stretchable Displays

High-resolution stretchable displays are an essential and rapidly advancing frontier in stretchable electronics, playing a pivotal role in wearable devices, electronic skins, and soft robotics. These displays must sustain excellent visual clarity and color fidelity, even under substantial mechanical deformation. Overcoming challenges such as strain-induced resolution loss, brightness degradation, and image distortion requires integrated hardware and software solutions.

On the hardware side, recent progress centers on innovative device architectures and advanced materials designed to maintain pixel density and display performance during mechanical strain. Yoo et al. [58] developed stretchable, high-resolution synesthesia displays that merge visual and acoustic signals, utilizing precise transfer printing on elastomeric substrates. These displays achieve over 120% stretchability and retain high performance after more than 5000 deformation cycles, enabling multisensory human–machine interaction beyond traditional visual feedback.

Lee et al. [59] introduced a novel device architecture known as the hidden active area (HAA), a three-dimensional pixel design in stretchable OLEDs where pixels are initially folded and hidden beneath the substrate but become active upon biaxial stretching. This design effectively resolves the common trade-off between stretchability and pixel fill factor, maintaining over 87% fill factor after 30% biaxial strain. Finite element method simulations and an optimized quadaxial stretching process ensure mechanical robustness and stable performance on complex curved surfaces.

Complementing these hardware innovations, software-based compensation strategies address image degradation due to mechanical strain. Min et al. [60] proposed strain-sensor-in-pixel (S-SIP) technology, embedding miniature strain sensors directly within pixel circuits. This enables real-time detection of local strain and dynamic pixel activation adjustments, effectively compensating for deformation-induced resolution loss. This approach was validated by extensive mechanical cycling and simulation, demonstrating promise for scalable, next-generation stretchable displays.

In addition, Jung et al. [61] developed a distribution density-aware compensation method (DDAM), leveraging human visual perception models to dynamically adjust pixel activation density based on stretch magnitude and viewing conditions. This algorithm significantly enhances brightness uniformity and image fidelity under varying deformation states, providing a perceptually informed software solution that complements the hardware advances.

Despite these considerable advances, challenges remain in improving power efficiency, manufacturing scalability, and long-term device reliability. In particular, scalable and manufacturable fabrication processes are essential to transition stretchable displays from laboratory demonstrations to real-world applications. Several promising approaches have been explored in recent years, including transfer printing, which enables the precise placement of micro-LEDs or OLED components onto stretchable substrates; and roll-to-roll (R2R) printing, which supports large-area, continuous fabrication. Other digital printing techniques, such as inkjet and screen printing, offer design flexibility and cost-effectiveness, particularly for wearable or disposable applications. While these methods show great potential, challenges remain in patterning resolution, alignment accuracy, and material compatibility under strain. Future research must integrate these scalable manufacturing approaches with ongoing hardware and software innovations to realize multifunctional, energy-efficient, and deployable stretchable displays for next-generation healthcare, wearable electronics, and soft robotics.

#### 3.2.2. Energy-Autonomous Systems

Energy-autonomous systems, which can harvest and store energy without reliance on external power sources, are critical enablers for the widespread adoption of wearable and stretchable electronics. These systems enhance device portability and usability by enabling continuous operation in diverse environments, addressing the practical challenges of battery limitations and frequent recharging.

A comprehensive overview of this rapidly evolving field is provided by Wu et al. [62], who reviewed a wide range of energy harvesters designed for wearable and stretchable electronics. Their analysis spans piezoelectric, thermoelectric, triboelectric, and photovoltaic mechanisms, highlighting numerous research efforts focused on enhancing energy conversion efficiency while maintaining mechanical stretchability. The review also explores emerging material systems and integration techniques that facilitate seamless embedding of energy devices into soft electronics.

In a complementary study, Yan et al. [63] surveyed the development of flexible and stretchable organic solar cells (OSCs), particularly focusing on transparent conductive electrodes, photoactive materials, and associated device architectures. Their work introduces various research initiatives aimed at improving mechanical robustness, energy output, and adaptability of OSCs for wearable applications, providing a valuable summary of state-of-the-art strategies and remaining technical hurdles.

**Figure 9:**
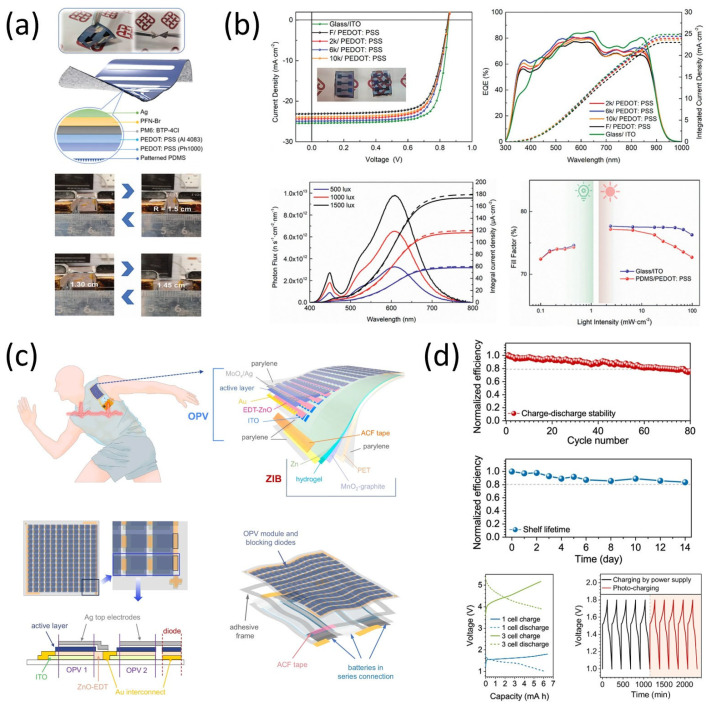
Energy-autonomous systems for wearable electronics. (**a**) Structure and photographs of stretchable organic solar cells based on ITO-free anti-reflection substrates. (**b**) Device performance under various illumination conditions, including current density–voltage characteristics and spectral response. Adapted with permission from Ref. [64]. © 2021 Wiley-VCH GmbH. (**c**) Schematic of an ultraflexible integrated energy harvesting–storage system with OPV modules, batteries, and power management on a stretchable substrate. (**d**) Electrical stability and charging performance of the system under mechanical deformation and light exposure. Reproduced from Ref. [65]. © 2024 The Authors. Licensed under CC BY 4.0.

Beyond these broad overviews, specific innovations continue to demonstrate the feasibility of practical energy-autonomous systems. Huang et al. [64] developed a stretchable, ITO-free organic solar cell incorporating a microtextured anti-reflection substrate. This design not only enhanced optical absorption and mechanical compliance but also delivered a power conversion efficiency of 15.3% under standard solar illumination and sustained efficient performance under indoor lighting (Figure 9a,b). These characteristics demonstrate its suitability for real-world, body-mounted energy harvesting.

Furthermore, Saifi et al. [65] reported an ultraflexible energy harvesting–storage platform that integrates organic photovoltaic (OPV) modules with zinc-ion batteries (ZIBs) on stretchable substrates. Their architecture includes energy harvesting, storage, and power management units fabricated on a soft parylene-based film (Figure 9c). Notably, the system demonstrated stable energy cycling and reliable powering of wearable electronics during human motion, validating its potential for self-sustained wearable operation (Figure 9d).

Despite notable progress, challenges remain in maximizing energy conversion, ensuring long-term device stability, and developing scalable fabrication methods. Future work must focus on multifunctional materials and unified device platforms that integrate harvesting, storage, and control to unlock the full potential of stretchable, autonomous electronics.

#### 3.2.3. Durable Encapsulation Techniques

Durable encapsulation is a critical factor for ensuring the long-term reliability and performance of stretchable electronic devices, especially stretchable OLEDs and displays that are exposed to environmental factors such as moisture, oxygen, and mechanical stresses. Robust encapsulation prevents degradation, prolongs device lifetime, and maintains optical and electrical performance under repeated mechanical deformation.

Recent advances have introduced various materials and techniques to enhance encapsulation stability. Liu et al. [66] reported thermal-radiation-assisted metal encapsulation methods that significantly improve the mechanical durability and electrical conductivity of conductive films, enabling stretchable devices to maintain stability under cyclic strain. Zhang et al. [67] developed stretchable PDMS hybrid films doped with SiO2 and enhanced by atomic layer infiltration, achieving outstanding water vapor barrier properties and mechanical reliability even after thousands of low-strain fatigue cycles. Their approach preserves the photoluminescence intensity of encapsulated quantum dots after prolonged exposure to harsh conditions, highlighting the potential for reliable stretchable encapsulation.

Shin et al. [68] investigated stoichiometric silicon nitride thin films as gas barrier layers for flexible and stretchable OLED encapsulation. By optimizing film composition via nitrogen neutral beam treatment, they achieved ultra-low water vapor transmission rates and demonstrated device reliability under harsh temperature and humidity conditions for extended periods.

In a more recent development, Kim et al. presented a robust dual-layer encapsulation strategy tailored for textile-based stretchable OLEDs, integrating a 30 nm nanolaminate of ALD-grown Al_2_O_3_/ZnO with a spin-coated parylene-C overlayer. The ALD nanolaminate served as an ultra-thin, conformal inorganic barrier, effectively minimizing water vapor and oxygen permeation, while the parylene-C topcoat provided mechanical flexibility, chemical resistance, and enhanced physical robustness. This bilayer encapsulation system demonstrated outstanding environmental stability, retaining over 90% of its initial luminance after more than 5000 dynamic folding cycles and multiple washing tests. Notably, the system maintained mechanical integrity and barrier function without visible delamination or microcracking, even under cyclic strain and high-humidity exposure. These results establish a promising path for reliable encapsulation in deformable and washable stretchable electronics, especially in applications requiring high durability on textile substrates [69].

Despite these promising developments, the current state of stretchable encapsulation technologies remains inadequate for applications requiring high tensile strains or long-term operation in harsh environments. Many existing solutions are limited by their elongation thresholds, susceptibility to delamination, or trade-offs between mechanical compliance and barrier performance. To address these challenges, future research must pivot toward the co-design of materials and encapsulation architectures. This includes hierarchical multilayer stacks combining ultrathin inorganic barriers (e.g., ALD-grown oxides) with elastomeric interlayers engineered for strain decoupling, as well as bio-inspired structures that mimic the stress-dissipating properties of skin or tendon. Additionally, dynamic or responsive encapsulation systems—capable of altering stiffness or permeability in response to mechanical strain—may offer adaptive protection under real-world usage conditions. 1qjsAchieving truly durable and scalable encapsulation will also require advances in interface engineering, including the development of low-modulus adhesive systems that maintain adhesion without inducing localized strain accumulation. Ultimately, a systems-level approach that integrates material science, mechanical modeling, and scalable fabrication will be essential to unlock the full potential of stretchable displays for commercial and biomedical applications.

#### 3.2.4. Integration with Artificial Intelligence

The integration of artificial intelligence (AI) with stretchable and wearable electronics is rapidly transforming the landscape of human–machine interfaces, health monitoring, and interactive devices. This synergy leverages AI’s powerful data processing and pattern recognition capabilities alongside the mechanical flexibility and conformability of stretchable devices, enabling intelligent systems that are both adaptable and unobtrusive.

Dong et al. [70] provide a comprehensive review of fiber- and fabric-based piezoelectric and triboelectric nanogenerators (NGs) designed for flexible and stretchable electronics. Their work highlights the potential of textile-integrated NGs as multifunctional self-powered sensors that enable continuous mechanical energy harvesting and sensing, crucial for powering AI-enabled wearable systems. However, they also emphasize challenges such as integrating NGs with textile manufacturing processes and ensuring both electrical performance and textile-related properties are simultaneously optimized.

**Figure 10:**
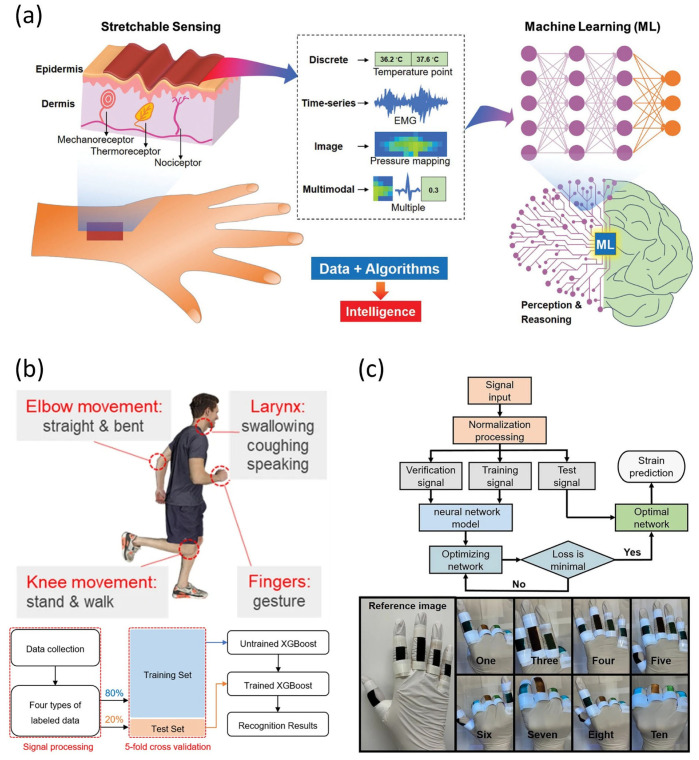
Research on the integration of artificial intelligence with stretchable sensing systems. (**a**) General framework of AI-enabled stretchable electronics, illustrating how discrete and multimodal biosignals are processed through ML models for intelligent applications. Adapted from Ref. [71]. © 2021 Wiley-VCH GmbH. Reproduced with permission. (**b**) Ultralight and biocompatible all-fiber motion sensor (AFMS) integrated with AI algorithms for motion monitoring and wearable electronics. Reproduced from Ref. [72]. © 2022 The Authors. Licensed under CC BY 4.0. (**c**) Conceptual design and implementation of a smart glove system using a sandwich-structured mechanoluminescent film and deep learning-based AI for high-accuracy hand gesture recognition. Reproduced from Ref. [73]. © 2025 The Authors. Licensed under CC BY 4.0.

Building on this foundation, Wang et al. [71] explore the fusion of stretchable sensing technologies with machine learning (ML) algorithms. They discuss recent advances in skin-like stretchable sensors that conform to complex, dynamic surfaces for high-quality biosignal acquisition. The authors underscore how ML enhances perception and reasoning tasks, facilitating sophisticated applications in human–machine interfaces, healthcare diagnostics, and robotics. This fusion represents a vital step towards autonomous, intelligent wearable systems (Figure 10a).

Lin et al. [72] present an ultralight, flexible, and biocompatible all-fiber motion sensor integrated with AI algorithms for wearable electronics. Their radial anisotropic porous silver fiber (RAPSF)-based sensor exhibits exceptional flexibility, breathability, and sensitivity to motion. By combining the sensor with machine learning-based classification, the system achieves over 85% accuracy in recognizing various human movement states such as walking and running, demonstrating its potential for motion tracking, gait analysis, and personalized healthcare applications (Figure 10b).

Finally, Dong et al. [73] report an AI-assisted flexible mechanoluminescent strain sensor system that integrates deep learning neural networks for rapid, accurate interpretation of light emission signals generated under mechanical strain. The sandwich-structured flexible mechanoluminescent film, combined with a color processing algorithm, enables fast and precise recognition of complex hand gestures. This innovative platform addresses traditional limitations of flexible strain sensors, accelerating the transition of intelligent wearable devices from laboratory research to practical consumer electronics (Figure 10c).

Building on these developments, more recent studies have explored deeper integration between AI and stretchable systems through physically embedded neuromorphic and edge-computing architectures. For instance, Liu et al. [74] introduced a stretchable neuromorphic tactile system that combines a strain-insensitive triboelectric nanogenerator (TENG) with a spiking artificial neuron circuit. Their device forms a 64 × 64 tactile matrix capable of real-time signal processing based on spike timing, demonstrating that neuromorphic computing can be directly implemented in soft, deformable substrates. While not a display device, this system exemplifies how intelligent sensing and perception can be embedded into stretchable electronic skins.

In a complementary direction, Liu et al. [75] developed a wearable in-sensor computing platform based on stretchable organic electrochemical transistors (OECTs). By incorporating supramolecular buffer layers, they achieved over 50% stretchability and enabled on-body acquisition and classification of biosignals—such as heart rate and skin conductance—using a compact, watch-compatible module. This approach bypasses external processors, highlighting the potential of localized AI inference within stretchable electronics. Although not directly related to display operation, such architectures open the door for future displays to respond adaptively to user physiology and context.

These recent studies are not display-centric per se, but they reflect a critical evolution in the field: the integration of AI functionality into mechanically compliant systems. They demonstrate how stretchable platforms can serve as intelligent, context-aware nodes that sense, process, and respond autonomously. Drawing from these insights, future stretchable display systems may evolve to incorporate AI-assisted luminance modulation, real-time strain compensation, and personalized interface adaptation, particularly through biosignal mapping and embedded neural processing. To realize such a vision, an integrated design strategy is essential, spanning material science, soft circuit architecture, biosignal transduction, and low-power AI algorithms. This convergence will mark the transition from passive stretchable displays to perceptive and adaptive display systems, enabling the next generation of wearable electronics that are not only flexible but also intelligent.

## 4. Conclusions

Stretchable display technology has experienced significant and rapid advancements over the past several years, driven by breakthroughs in novel materials, innovative device architectures, and sophisticated system-level integrations. This comprehensive review has covered essential developments spanning intrinsic stretchable displays, wavy surface configurations, and hybrid approaches, each presenting distinct challenges and opportunities. The discussion of diverse applications—including wearable healthcare devices, smart textiles, implantable biomedical systems, and soft robotics—demonstrates the transformative potential of stretchable displays in enabling next-generation human–machine interfaces that seamlessly blend with the human body and dynamic environments.

Significant progress has been made toward achieving high-resolution stretchable displays capable of maintaining exceptional optical performance despite mechanical strain. This has been complemented by advances in energy-autonomous systems, which enable self-powered operation critical for untethered wearable devices, and durable encapsulation techniques that ensure long-term device stability and protection against environmental factors such as moisture and oxygen. Furthermore, the integration of artificial intelligence with stretchable electronics has opened new horizons in device functionality, providing intelligent sensing, real-time adaptive responses, and enhanced user interaction tailored to complex physiological and environmental conditions.

Nevertheless, despite these strides, several formidable challenges remain. Enhancing power efficiency remains a top priority to extend the operating time of wearable and implantable systems without increasing device size or weight. Improving large-scale manufacturability is essential for transitioning from laboratory prototypes to commercially viable products, demanding cost-effective and reproducible fabrication methods. Ensuring long-term reliability under repeated mechanical deformation and environmental exposure is critical for practical deployment. Additionally, achieving a delicate balance between mechanical flexibility, stretchability, and robust environmental protection continues to be an engineering and materials science challenge.

Looking ahead, future research must focus on the synergistic integration of hardware innovations—such as advanced materials, novel device designs, and encapsulation strategies—with software intelligence, including adaptive algorithms and artificial intelligence. This multidisciplinary approach is vital for developing multifunctional, scalable, and robust stretchable display systems that can meet the complex demands of real-world applications. The convergence of these efforts will likely spur breakthroughs that enable truly seamless human–machine interfaces with applications extending from personalized healthcare and smart textiles to immersive augmented reality and soft robotics.

In summary, stretchable display technology stands at the forefront of the flexible electronics revolution, poised to fundamentally reshape a broad spectrum of industries. Continued interdisciplinary collaboration, innovation, and focused research are essential to overcome remaining obstacles and unlock the full potential of stretchable displays, bringing them from cutting-edge research into widespread practical use in everyday life.

## Figures and Tables

**Table 1:** Summary of representative stretchable display technologies categorized by structural approach and specific sub-strategies within hybrid systems.

Approach	Subcategory	Mechanism	Typical Materials/ Structures	Stretchability	Key Features
Intrinsic	–	All functional components (electrodes, substrates, emissive layers) are intrinsically stretchable	AgNW, CNT, PEDOT:PSS, PU, SEBS, stretchable polymer LEDs	~20–50%	Fully compliant systems, simple integration, limited resolution
Wavy Surface	–	Out-of-plane strain relief via pre-straining, buckling, or surface wrinkling	Buckled inorganic thin films, wrinkled metal/organic layers, elastomer substrates	~30–100%	High stretchability, tunable geometry, effective stress redistribution
Hybrid	(1) Interconnect-focused (Serpentine)	Mechanical strain is absorbed through serpentine-shaped metal interconnects	Cu, Au, Ag serpentine wires, CNT-based stretchable circuits	>100%	Excellent durability, electrical stability, requires larger layout area
	(2) Device-integrated (Island–Bridge)	Rigid electronic islands connected by soft bridges to isolate strain	µLED, OLED islands + liquid metal, nanomesh, or serpentine bridges	~20–40%	Enables high-resolution displays, stable under strain, requires precise alignment
